# Enhanced Mechanical Properties and Isotropy of Mg-2Al-0.8Sn Alloy through Ca Addition

**DOI:** 10.3390/ma14247557

**Published:** 2021-12-09

**Authors:** Yuan Miao, Chao Wang, Minghui Wang, Hai Deng, Pinkui Ma, Zhigang Li

**Affiliations:** 1 Key Laboratory of Automotive Materials Ministry of Education, School of Material Science and Technology, Jilin University, Changchun 130022, China; miaoyuan19@mails.jlu.edu.cn (Y.M.); minghui@jlu.edu.cn (M.W.); mapk@jlu.edu.cn (P.M.); 2 National Railway Vehicle Engineering Research Center, CRRC Changchun Railway Vehicles Co., Ltd., Changchun 130062, China; denghai@cccar.com.cn; 3 State Key Laboratory of Automotive Simulation and Control, Jilin University, Changchun 130022, China

**Keywords:** Mg-Al-Sn alloy, Ca content, microstructure, anisotropy, tensile properties

## Abstract

Calcium (Ca), with abundant and cheap reserves, is a potential element to facilitate the further application of Mg-Al-Sn based alloys. Here, effects of Ca content on the microstructure and tensile properties of Mg-2.0Al-0.8Sn (wt.%) alloys were systematically studied. The experimental results illustrated that the strength, ductility and isotropy of the alloys improved simultaneously with the increase of Ca content. The better ductility and isotropy could be contributed to the weakened texture via particle stimulation nucleation mechanism. The higher strength benefited from the combination of finer grains, more precipitates and residual dislocation density. Eventually, the Mg-2.0Al-0.8Sn-0.5Ca (wt.%) alloy showed the best room-temperature balance of strength and ductility with a yield strength of ∼226.0 MPa, an ultimate tensile strength of ∼282.4 MPa and a fracture elongation of ∼20.2%, which has huge potential as an applicable low-cost high-performance magnesium alloy.

## 1. Introduction

Magnesium (Mg) and its alloys are hoped to tremendously reduce the mass of vehicles, allowing for better fuel efficiency and lower CO_2_ emissions [1,2,3]. However, they have poor mechanical properties at room temperature, which make for a huge challenge to wide application [4,5]. In the past few decades, people have made attempts to improve the strength and ductility via tailoring a Mg matrix with added alloying elements. The combined effect of adding Al and Sn has been reported to be of great help to ductility improvement by reducing the critical resolved shear stress (CRSS) difference between non-basal slip and basal slip [6,7]. Hence, Mg-Al-Sn based (AT system) alloys have received much attention for their potentially excellent mechanical properties [8].

Aiming at further development and application of AT system alloys, it is worth trying to enhance the mechanical properties of the alloy through alloying. As an element with abundant and cheap reserves, Ca can randomize the texture of Mg alloys and form precipitates in Sn-containing Mg alloys because of its large electronegativity difference with Sn [9,10]. Moreover, Ca can also form thermally stable second phases, Al_2_Ca and (Mg, Al)_2_Ca, with Al, which are affiliated with the ratio of Al and Ca content [11,12]. Based on the above reasons, a great deal of studies have proved that Ca could boost the tensile properties of Mg-Sn alloy and Mg-Al alloy. Pan et al. [13] designed a Mg-2Sn-2Ca alloy (wt.%) with extraordinary yield strength (YS, ~360 MPa), depending on its submicron matrix grains (~0.32 μm) and Mg_2_Ca precipitates. By adjusting the Al/Ca ratio, Li [11] et al. designed a high-strength Mg-2.7Al-3.5Ca-0.4Mn (wt.%) alloy with a ultra-high strength (YS: 438 MPa, ultimate tensile strength (UTS): 457 MPa), and a large-ductility Mg-4.4Al-1.1Ca-0.4Mn (wt.%) alloy with an elongation (EL) of 29.9%. Therefore, Ca is also a potential element to improve the mechanical properties of AT system alloys at room temperature.

However, the detailed roles of Ca content on the Mg-Al-Sn alloys have not been clarified. Thus, in this study, Mg-Al-Sn alloys with different Ca content were cast, rolled and annealed. In addition, we primarily studied the tensile properties and asymmetry of as-annealed Mg-2.0Al-0.8Sn-xCa (x = 0.0, 0.3, 0.5 wt.%) alloys. Such research will offer an essential basis for further boosting the mechanical properties and practical applications of AT system Mg alloys.

## 2. Experimental Procedure

**Figure 1:**
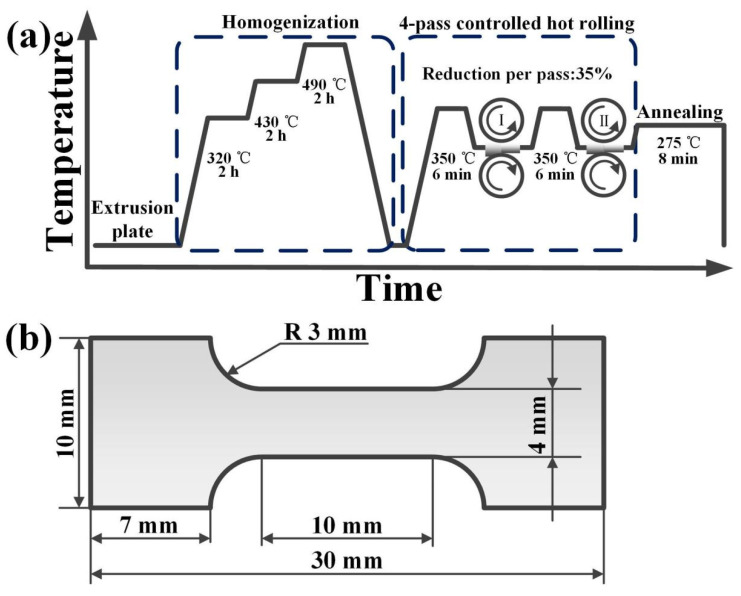
Schematic representation of (**a**) the preparation process and (**b**) the tensile test specimen.

In this work, Mg-2.0Al-0.8Sn-xCa (x = 0, 0.3 and 0.5 wt.%) alloys were fabricated by melting pure Mg (99.90 wt.%), pure Al (99.90 wt.%), pure Sn (99.90 wt.%) and Mg–25Ca (wt.%) in an electric furnace under a protective gas mixture of 99.5% CO_2_ and 0.5% SF_6_ at ~953 K. After casting, ingots were extruded at 653–703 K, with a ram speed of about 19.3 −23.2 mm s^−1^ and an extrusion ratio of 28. The extruded plates were then homogenized at 320 °C, 420 °C and 490 °C for 2 h, and hot-rolled at 350 °C from ~5 mm to ~0.9 mm by 4 passes, with ~35% reduction per pass. During the whole rolling process, the rollers were maintained at 100 °C and the rolling samples were preserved for 6 min between passes. Afterward, the sheets were annealed at 275 °C for 8 min. The preparation process is shown in Figure 1a, and the nominal compositions of the prepared alloys are presented in Table 1. For simplicity, the as-annealed samples are denoted below as AT21, ATX2103 and ATX2105.

The microstructure was characterized by a scanning electron microscope (SEM, VEGA 3 XMU, TECAN Czech, Mannedorf, Switzerland) equipped with energy dispersive spectrometer (EDS, OXFORD X-Max^N^ AZtec) and electron backscatter diffraction (EBSD) detector (OXFORD NordlysNano AZtec). The analysis on phase constituents was performed by X-ray diffraction (XRD, Model D Max 2500PC Rigaku Tokyo, Japan). A transmission electron microscope (TEM, JEM-2100F, JEOL Ltd., Tokyo, Japan) was used to explore the information of nanoscale precipitates. TEM samples were thinned via mechanical grinding combined with ion-beam thinning.

All the dog-bone shaped tensile samples (30 mm × 10 mm) were cut from the annealed sheets along rolling direction (RD) according to the cutting scheme shown in Figure 1b. Tensile tests were conducted through the universal test machine (SHIMADZU, Suzhou, China) under a strain rate of 1.0 × 10^−3^ s^−1^. A minimum of five samples were tested to ensure reliability.

## 3. Results and Discussion

### 3.1. Microstructural Characteristics

**Figure 2:**
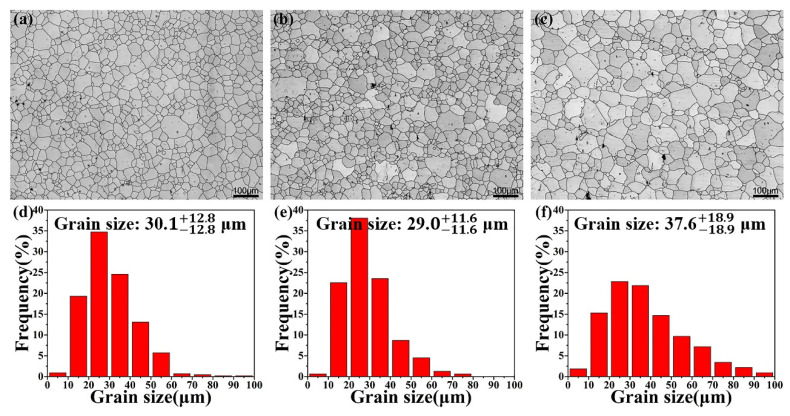
EBSD maps and corresponding grain size distributions of as-homogenized (**a**,**d**) AT21, (**b**,**e**) ATX2103 and (**c**,**f**) ATX2105 alloys.

The EBSD maps and grain size distribution maps of the as-homogenized samples are shown in Figure 2. The average grain sizes of as-homogenized AT21, ATX2103 and ATX2105 alloys are ~30.1 μm, ~29.0 μm and ~37.6 μm, respectively. All samples have large grain sizes and no regular changes in size, indicating that the initial structure has a negligible effect on the properties after rolling.

**Figure 3:**
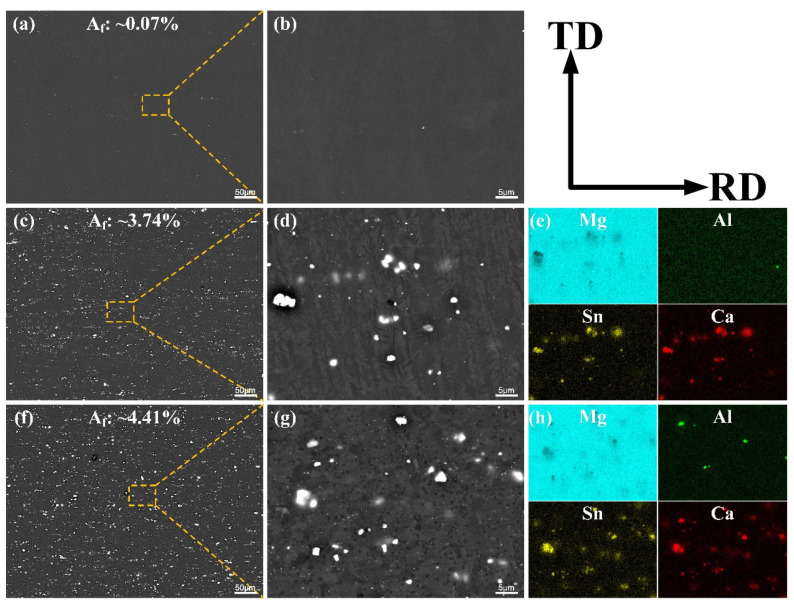
The backscattered electron SEM micrographs of (**a**,**b**) AT21, (**c**,**d**) ATX2103 and (**f**,**g**) ATX2105 alloys; EDS micrographs of (**e**) ATX2103 and (**h**) ATX2105 alloys.

**Figure 4:**
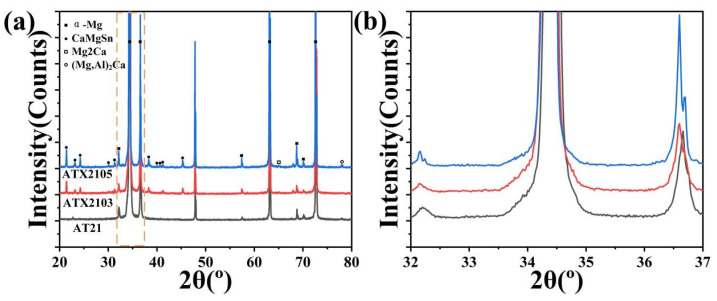
(**a**) The XRD patterns of the AT21, ATX2103 and ATX2105 samples, and (**b**) the enlarged section of the XRD patterns.

**Figure 5:**
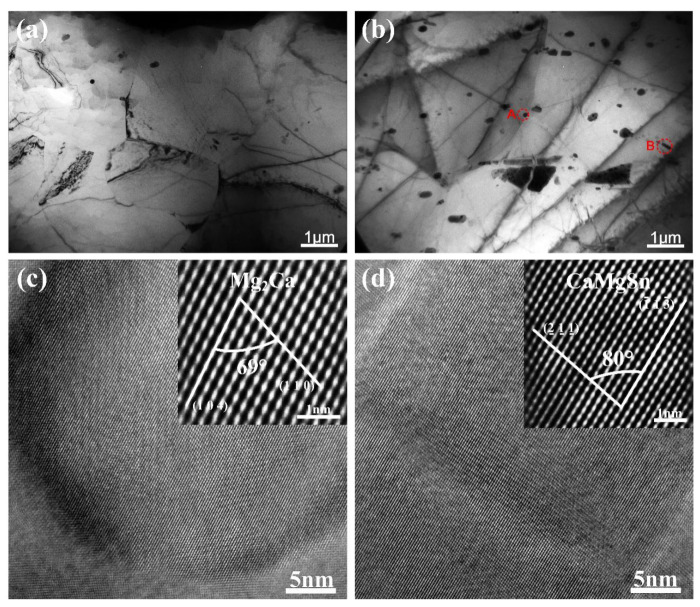
TEM micrographs of (**a**) ATX2103 and (**b**) ATX2105; the HRTEM micrograph of (**c**) particle A and (**d**) particle B.

Figure 3 presents the SEM and corresponding EDS results of as-annealed AT21, ATX2103 and ATX2105 alloys. With Ca content increasing from 0.3 to 0.5 wt.%, there is an increasement in the area fraction of phases (varying from ~3.74% to 4.41%) but almost no second phase in the AT21 sample. Combining XRD maps with EDS micrographs (Figure 4), it can be concluded that massive irregular particles in ATX2103 and ATX2105 alloys are CaMgSn phase and the Al-containing particles are (Mg, Al)_2_Ca. In addition, the relative intensities of diffraction peaks for the CaMgSn and (Mg, Al)_2_Ca phases increase with a rise in Ca content from 0.3 to 0.5 wt.%, indicating that the volume fraction of them increases accordingly. To study the characteristics of precipitates, which are too small to be analyzed by SEM, TEM analysis was conducted on the annealed alloys (Figure 5). In ATX2105 alloy (Figure 5b), there exists more spherical fine precipitates than in ATX2103, displaying the volume fractions of precipitates of ~1.40 × 10^−2^ and ~0.49 × 10^−2^, respectively. The precipitation of ATX2103 alloy is basically spherical, while ATX2105 also has a rod-like phase. Through HRTEM analysis of particles A and B (Figure 5c,d), it is concluded that the spherical particles are Mg_2_Ca and the rod-shaped particles are CaMgSn phase.

**Figure 6:**
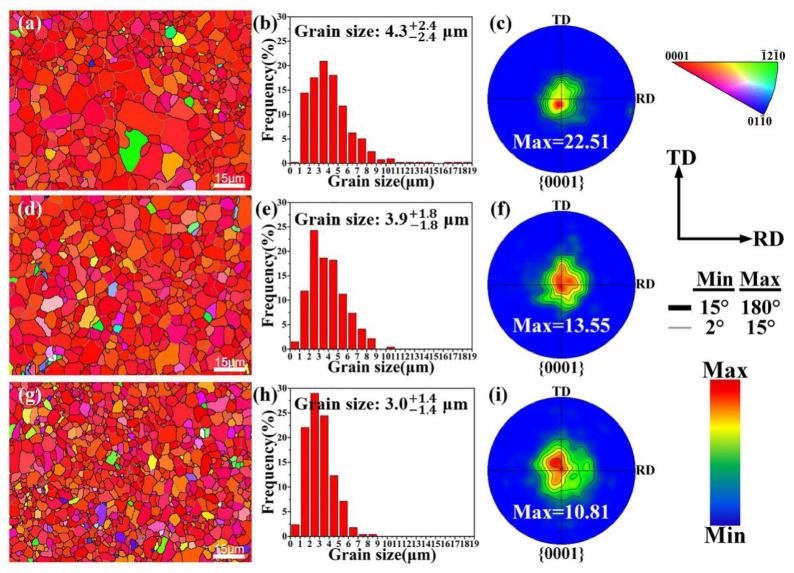
Inverse pole figure (IPF) maps with corresponding grain size distributions and {0001} pole figures of (**a**–**c**) AT21, (**d**–**f**) ATX2103 and (**g**–**i**) ATX2105 alloys.

**Figure 7:**
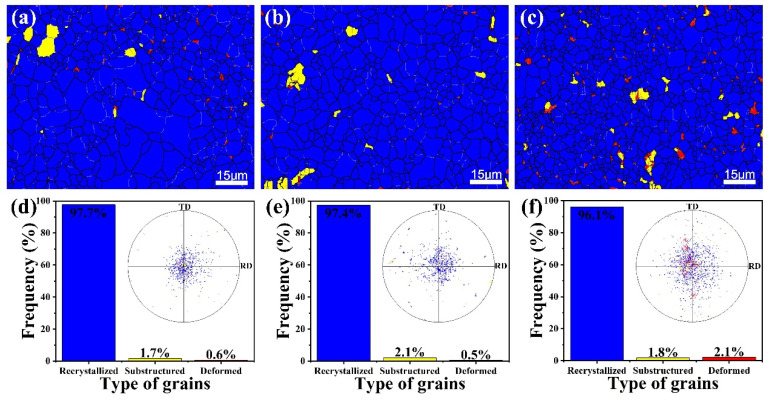
Different types of grains of (**a**) AT21, (**b**) ATX2103 and (**c**) ATX2105 alloys; (**d**–**f**) frequency and corresponding (0001) pole figures of the different types of grains shown in (**a**–**c**).

The microstructures and the corresponding grain size distributions of the annealed alloys are presented in Figure 6. For Ca modification, there exists a distinct grain refinement from ~4.3 μm to ~3.0 μm, which is mainly due to the restraining effect on the growth of grains from precipitated particles [14,15]. In terms of texture, alloys displayed typical basal textures, while the texture intensity was apparently weakened by the addition of Ca. Previous studies have reported that coarse particles larger than 1 μm in size can trigger the particle stimulation nucleation (PSN) mechanism, reduce the orientation correlation between recrystallization grains and present grains, and thus weaken the texture [16,17,18]. It is noteworthy that Ca-modified alloys contain particles larger than 1 μm in size, and the basal poles of recrystallized grains disperse away from the normal direction more so than those of substructured and deformed grains (Figure 7). Determining PSN mechanism is the key factor to weaken texture [19].

### 3.2. Mechanical Properties

**Figure 8:**
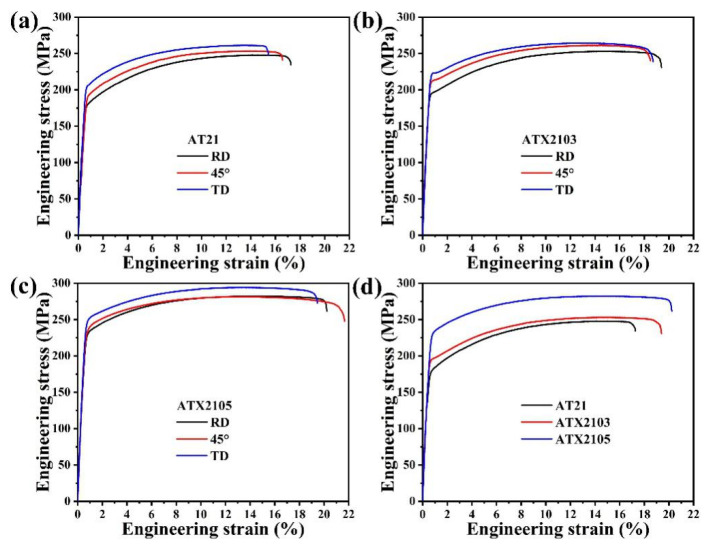
Engineering stress–strain curves of (**a**) AT21, (**b**) ATX2103 and (**c**) ATX2105; (**d**) the comparison of tensile properties of the samples tensiled along RD.

Tensile engineering stress–strain curves for AT system alloys stretching along the RD, 45°, and TD, respectively, are revealed in Figure 8a–c, and related YS, UTS and EL are presented in Table 2. As is observed in Figure 8, both the strength and the EL increase monotonously with increasing of Ca content. And ATX2105 possesses the best tensile properties with a YS of ~226.0 MPa, an UTS of ~282.4 MPa and an EL of ~20.2% when tensiled along RD. It is evident that all samples display tensile property anisotropy. The minimum and maximum YS differences of AT21, ATX2103 and ATX2105 are 34.1, 29.8 and 17.6 MPa, respectively, which indicates that the anisotropy becomes weaker with increasing of Ca content. 

**Figure 9:**
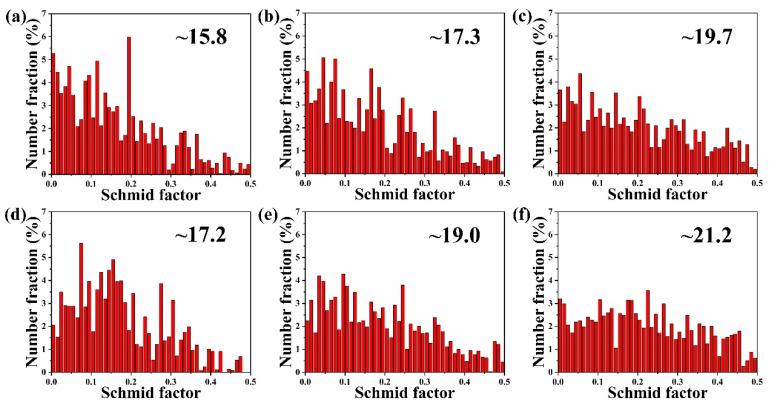
Schmid factor distribution maps based on {0001} 〈110〉 slip system for RD of (**a**) AT21, (**b**) ATX2103 and (**c**) ATX2105, and for TD of (**d**) AT21, (**e**) ATX2103 and (**f**) ATX2105, respectively.

Figure 9 shows the Schmid factor (SF) distributions of the {0001} 〈110〉 slip system of all sheets along the RD and TD. Usually, the SF closely related to the grain orientation, and the larger the SF, the more favorable the orientation is for basal slip [20]. With the increase of Ca content, SF obviously increases, and the difference of SFs along different directions becomes smaller. This indicates that the addition of Ca promotes the basal slip and decreases the difference of activated slips for different directions, resulting in the higher ductility and weaker anisotropy of the Ca modified samples [21]. 

The YSs of all samples result from the synergetic role of fine grains, tiny precipitates, solute atoms, and residual dislocation density [22]. The contribution of fine grains ($\sigma_{grain}$) can be determined by the Hall–Petch relationship [23]:

(1) $$\sigma_{grain}=kd^{-\frac{1}{2}}\;\;\;\;\;\;\;\;$$

where k is the Hall–Petch coefficient of 290 MPa ${\mu m}^{1/2}$ and d is the average grain size [24]. On the basis of the measured average grain sizes of the alloys, the fine-grain contributions to the YSs are ~139.8 MPa, ~146.8 MPa and ~167.4 MPa.

Precipitation strengthening ($\sigma_{p}$) can be evaluated by the Orowan looping mechanism. Since there is almost no second phase in AT21, precipitation strengthening is considered to be the only strengthening mechanism of Ca-containing samples. In addition, the increments in YS due to tiny precipitates strengthening are expressed as [25]:

(2) $$\sigma_{p}=M\frac{Gb}{2\pi \sqrt{1-v}\left(\frac{0.953}{\sqrt{f}}-1\right)d_{t}}ln\frac{d_{t}}{b}\;\;\;\;\;\;\;$$

where M, G, b and v are the Taylor factor, shear modulus, Burgers vector and Poisson’s ratio (3.06 [26]), 1.66 × 10^4^ MPa [26], 3.2 × 10^−10^ m [26] and 0.35 [26] for Mg), and $d_{t}$ and f are the mean diameter and volume fraction of precipitates (estimated to be 127 nm and 0.4 × 10^−2^ for ATX2103, 145 nm and 1.4 × 10^−2^ for ATX2105). According to the equation, a smaller particle size and a higher volume fraction can lead to a more significant strengthening effect on alloys. Additionally, the calculation contributions of precipitation strengthening in both alloys are ~10.7 MPa and ~19.2 MPa.

In the studied alloys, Al, Sn, and Ca atoms may have a solid solution strengthening effect. However, owing to the similar atomic radius of Sn and Mg atoms, the solid solution strengthening effect of Sn can be negligible [22]. It has been reported that in Sn-containing Mg alloys, Ca formed the second phase, causing the solid solution strengthening effect of Ca to be weakened [27,28]. The XRD pattern between 32° and 37° (Figure 4b) further shows that the α-Mg diffraction peaks have almost no angular shift with increasing of Ca content, indicating that the solid solution strengthening effect of Ca can also be ignored in this alloy system [29,30]. Therefore, the strengthening contribution from solute atoms can be estimated by the following formula [31]:

(3) σss=σAl=kAl1nCAl

where n is a constant of 2/3 [4], $k_{Al}$ is the constant for solute Al (196 MPa (at.%)^−2/3^ [22]), $C_{Al}$ is the concentration of solute Al, which is 1.82 at.% of the three alloys, and the calculated value is ~13.6 MPa.

**Figure 10:**
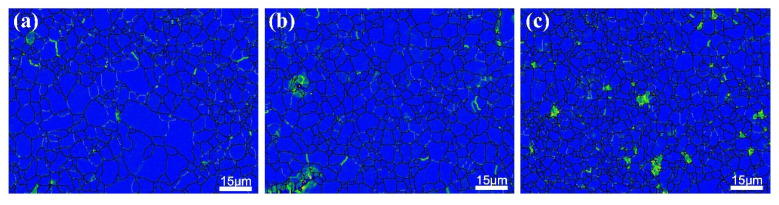
KAM results of (**a**) AT21, (**b**) ATX2103 and (**c**) ATX2105, respectively.

The dislocation strengthening depends on the dislocation density. The geometrically necessary dislocation (GND) density and the hardening contribution can be estimated by the equations as follow [27,32]:

(4) $$\rho_{GND}=\frac{2\theta }{\mu b}\;\;\;\;\;\;$$

(5) $$\sigma_{D}=M\alpha Gb\sqrt{\rho_{GND}}\;\;$$

where θ is the average misorientation, obtained from the Kernel average misorientation (KAM) dates (Figure 10); μ is the kernel size of 0.7 μm in our work. The predicted results of the dislocation strengthening are thus ~23.2 MPa, ~24.8 MPa and ~25.8 MPa.

The calculated contributions of various strengthening mechanisms are included in Table 3. It can be seen that the calculated YSs are close to the measured YSs, indicating that the calculated values are accurate. The increase in YS under the addition of Ca may be thanks to the improved grain refinement, precipitation and dislocation strengthening effects.

## 4. Conclusions

(1) With the addition of Ca in Mg-2.0Al-0.8Sn alloy from 0.0 wt.% to 0.5 wt.%, the strength, ductility and isotropy of the alloys increase simultaneously. In addition, the ATX2105 alloy shows the best tensile properties with a YS of ∼226.0 MPa, a UTS of ∼282.4 MPa and an EL of ∼20.2% along RD at room temperature.
(2) The second phases of the Ca-modified alloys are CaMgSn, (Mg, Al)_2_Ca phase and tiny Mg_2_Ca phase. However, when the Ca content is only 0.3 wt.%, there is only a small amount of (Mg, Al)_2_Ca and Mg_2_Ca. The AT21 alloy basically has no second phase.
(3) The enhancement of ductility and anisotropy via (0.0–0.5 wt.%) Ca addition is mainly attributed to the texture modification, which promotes the basal slip and decreases the differences between activated slips for different directions.
(4) The increase in YS under the addition of Ca originates from the synergetic effect of finer grain size, more tiny precipitates and residual dislocation density.

## Figures and Tables

**Table 1:** Compositions of Mg-2.0Al-0.8Sn-*x*Ca (*x* = 0.0, 0.3 and 0.5 wt.%) alloys.

Nominal Composition	Measured Composition (wt.%)			
	Al	Sn	Ca	Mg
Mg-2.0Al-0.8Sn-0.0Ca	1.96	0.77	-	Bal.
Mg-2.0Al-0.8Sn-0.3Ca	1.98	0.83	0.28	Bal.
Mg-2.0Al-0.8Sn-0.5Ca	2.01	0.79	0.51	Bal.

**Table 2:** Tensile properties of Mg-2.0Al-0.8Sn-xCa (x = 0, 0.3 and 0.5 wt.%) alloys.

		YS (MPa)	UTS (MPa)	EL (%)
AT21	RD	$171.0_{-0.4}^{+3.4}$	$248.0_{-3.6}^{+0.3}$	$17.3_{-2.3}^{+1.4}$
	45°	$193.5_{-2.7}^{+2.6}$	$253.4_{-2.1}^{+1.4}$	$16.6_{-2.2}^{+1.9}$
	TD	$205.1_{-3.5}^{+2.1}$	$261.3_{-2.2}^{+3.1}$	$15.4_{-1.3}^{+1.2}$
ATX2103	RD	$192.4_{-1.8}^{+2.2}$	$253.1_{-2.7}^{+1.1}$	$19.4_{-1.2}^{+1.1}$
	45°	$211.0_{-2.4}^{+3.2}$	$261.3_{-1.3}^{+2.2}$	$18.5_{-1.2}^{+2.3}$
	TD	$222.2_{-1.8}^{+2.1}$	$264.4_{-1.2}^{+3.0}$	$18.7_{-0.7}^{+0.2}$
ATX2105	RD	$226.0_{-0.7}^{+2.1}$	$282.4_{-1.9}^{+1.5}$	$20.2_{-0.3}^{+0.4}$
	45°	$230.0_{-1.6}^{+2.0}$	$281.5_{-1.8}^{+1.1}$	$21.7_{-1.6}^{+0.7}$
	TD	$243.8_{-1.0}^{+1.3}$	$294.3_{-1.2}^{+1.6}$	$19.5_{-0.4}^{+1.4}$

**Table 3:** Contributions of various strengthening mechanism into the YS of AT21, ATX2103 and ATX2105 alloys.

Nominal Composition	$\sigma_{grain}/\mathrm{MPa}$	$\sigma_{p}/\mathrm{MPa}$	$\sigma_{ss}/\mathrm{MPa}$	$\sigma_{D}/\mathrm{MPa}$	Calculated YS/MPa	Measured YS/MPa	Deviation/MPa
AT21	139.8	0	13.6	23.2	176.6	171.0	−5.5
ATX2103	146.8	10.7	13.6	24.8	195.6	192.4	−3.2
ATX2105	167.4	19.2	13.6	25.8	226.0	226.0	0

## Data Availability

Data sharing not applicable. No new data were created or analyzed in this study. Data sharing is not applicable to this article.
